# Conditional loss of *Brca1* in oocytes causes reduced litter size, ovarian reserve depletion and impaired oocyte *in vitro* maturation with advanced reproductive age in mice

**DOI:** 10.1016/j.ebiom.2024.105262

**Published:** 2024-07-30

**Authors:** Amy L. Winship, Lauren R. Alesi, Jessica M. Stringer, Yujie Cao, Yasmin M. Lewis, Lisa Tu, Elyse O.K. Swindells, Saranya Giridharan, Xuebi Cai, Meaghan J. Griffiths, Nadeen Zerafa, Leslie Gilham, Martha Hickey, Karla J. Hutt

**Affiliations:** a Monash Biomedicine Discovery Institute, Department of Anatomy and Developmental Biology, Development and Stem Cells Program, Monash University, Clayton, VIC, Australia; b University of Edinburgh, MRC Centre for Reproductive Health, Queens Medical Research Institute, Edinburgh, UK; c Breast Cancer Network Australia and Breast Cancer Trials Australia, Camberwell, VIC, Australia; d Gynaecology Research Centre, Royal Women's Hospital, Parkville, VIC, Australia; e Department of Obstetrics and Gynaecology, The University of Melbourne, Parkville, VIC, Australia

**Keywords:** Ageing, Primordial follicle, Oocyte, DNA repair, BRCA1, AMH

## Abstract

**Background:**

An estimated 1 in 350 women carry germline *BRCA1/2* mutations, which confer an increased risk of developing breast and ovarian cancer, and may also contribute to subfertility. All mature, sex steroid-producing ovarian follicles are drawn from the pool of non-renewable primordial follicles, termed the ‘ovarian reserve’. The clinical implications of early ovarian reserve exhaustion extend beyond infertility, to include the long-term adverse health consequences of loss of endocrine function and premature menopause. We aimed to determine whether conditional loss of *Brca1* in oocytes impacts ovarian follicle numbers, oocyte quality and fertility in mice with advancing maternal age. We also aimed to determine the utility of AMH as a marker of ovarian function, by assessing circulating AMH levels in mice and women with *BRCA1/2* mutations, and correlating this with ovarian follicle counts.

**Methods:**

In this study, we addressed a longstanding question in the field regarding the functional consequences of BRCA1 inactivation in oocytes. To recapitulate loss of BRCA1 protein function in oocytes, we generated mice with conditional gene deletion of *Brca1* in oocytes using *Gdf9*-Cre recombinase (WT: *Brca1*^fl/fl^*Gdf9*^+/+^; cKO: *Brca1*^fl/fl^*Gdf9*^cre/+^).

**Findings:**

While the length of the fertile lifespan was not altered between groups after a comprehensive breeding trial, conditional loss of *Brca1* in oocytes led to reduced litter size in female mice. *Brca1* cKO animals had a reduced ovarian reserve and oocyte maturation was impaired with advanced maternal age at postnatal day (PN)300, compared to WT animals. Serum anti-Müllerian hormone (AMH) concentrations (the gold-standard indirect marker of the ovarian reserve used in clinical practice) were not predictive of reduced primordial follicle number in *Brca1* cKO mice versus WT. Furthermore, we found no correlation between follicle number or density and serum AMH concentrations in matched samples from a small cohort of premenopausal women with *BRCA1/2* mutations.

**Interpretation:**

Together, our data demonstrate that BRCA1 is a key regulator of oocyte number and quality in females and suggest that caution should be used in relying on AMH as a reliable marker of the ovarian reserve in this context.

**Funding:**

This work was made possible through Victorian State Government Operational Infrastructure Support and Australian Government NHMRC IRIISS. This work was supported by funding from the 10.13039/501100000923Australian Research Council (ALW - DE21010037 and KJH - FT190100265), as well as the 10.13039/501100001026National Breast Cancer Foundation (IIRS-22-092) awarded to ALW and KJH. LRA, YML, LT, EOKS and MG were supported by Australian Government Research Training Program Scholarships. LRA, YML and LT were also supported by a Monash Graduate Excellence Scholarship. YC, SG and XC were supported by 10.13039/501100020821Monash Biomedicine Discovery Institute PhD Scholarships. LRA was also supported by a 10.13039/501100001779Monash University ECPF24-6809920940 Fellowship. JMS was supported by 10.13039/501100000925NHMRC funding (2011299). MH was supported by an 10.13039/501100000925NHMRC Investigator Grant (1193838).

**Research in context**

**Evidence before this study**

In mutant mice heterozygous for *Brca1*^+/Δ11^, a model in which exon 11 was globally deleted and the *Brca1*^Δ11^ gene product has compromised function, fewer primordial follicles were reported to be endowed in ovaries of female mice. Genetic deletion culminating in complete loss of BRCA1 protein function, specifically in postnatal oocytes has not been studied.

**Added value of this study**

This study comprehensively assessed the ovarian reserve of mice with a conditional knockout of *Brca1* in oocytes across the reproductive lifespan, ranging from PN5 to PN300, using females derived from multiple litters for every parameter assessed. The data demonstrate that conditional loss of *Brca1* in oocytes leads to reduced litter size, and a decline in the ovarian reserve and impaired maturation potential of oocytes with advanced maternal age.

**Implications of all the available evidence**

Given the critical role of the BRCA proteins in mediating DNA repair, our data extend the current knowledge of the quality control mechanisms in the female germline, in both primordial and mature oocytes and lay the foundations for future studies to test the specific effects of genotoxic insults on oocytes in context of loss of BRCA1.

## Introduction

The ovarian reserve comprises a pool of non-renewable primordial follicles, from which all growing, sex steroid-producing follicles and mature, ovulatory oocytes are drawn across the fertile lifespan.^1^^,^^2^ The size of the ovarian reserve is thought to be set by the time of birth and then diminishes gradually with age, until the loss of fertility and onset of menopause later in life.^3^ Ovarian reserve depletion can be accelerated by exposure to genotoxic insults (reviewed^4^, ^5^, ^6^), or genetic factors that may induce premature ovarian insufficiency (POI).^7^^,^^8^ The clinical implications of POI extend beyond infertility, including onset of premature menopause which is associated with increased risk of cardiovascular disease, osteoporosis, cognitive dysfunction and mental health disorders, among numerous others.^9^ Therefore, understanding the factors which regulate oocyte number and quality throughout ageing is of critical importance.

Multiple large-scale genome-wide association studies (GWAS) performed to isolate genetic determinants of age at natural menopause strongly implicate DNA repair as a primary biological pathway regulating reproductive senescence.^7^^,^^8^ The BRCA1 and BRCA2 proteins are key mediators of the homologous recombination DNA repair pathway, responsible for restoring DNA integrity after double-strand breaks (DSBs) are sustained. Whilst they have distinct functional roles in DNA repair processes, variants of both *BRCA1* and *BRCA2* that result in abrogated function of the encoded proteins confer a high risk of breast and ovarian cancer between the ages of 20–40 years, often before women have completed childbearing.^10^, ^11^, ^12^ Current international guidelines recommend that *BRCA1/2* mutation carriers undergo bilateral salpingo-oophorectomy (RRBSO) to decrease cancer risk before the average age of natural menopause (51 years).^13^ *BRCA1* mutation carriers must therefore make considered reproductive decisions, which is hampered by a lack of direct biomarkers of primordial follicles to predict the fertile lifespan in women. Serum anti-Müllerian hormone (AMH) concentrations are widely used clinically as an indirect marker of the ovarian reserve, even though AMH is not produced by primordial follicles.

The reproductive choices of *BRCA* mutation carriers are further complicated by longstanding uncertainty of whether or not they are at increased risk of infertility compared to non-mutation carriers,^14^ due to conflicting clinical data^15^ and limitations in assessing the ovarian reserve in women. Currently, it remains postulated that a reduced ability to repair DNA DSBs in *BRCA* mutation carriers may increase oocyte apoptosis, deplete the ovarian reserve and thereby accelerate ovarian ageing during reproductive life,^16^^,^^17^ but this remains to be determined unequivocally in an *in vivo* setting.

In a seminal study in 2013 that sought to address this experimentally using a preclinical model, fewer primordial follicles were reported to be endowed in ovaries of mutant mice heterozygous for *Brca1*^+/Δ11^, a model in which exon 11 was globally deleted and the *Brca1*^Δ11^ gene product has compromised function.^18^ Notably, since DNA DSBs are induced endogenously during prenatal follicle formation in the process of meiotic crossover and recombination,^4^^,^^19^ the findings from this model highlighted a fundamental role for BRCA1 in DNA DSB repair during follicle development. The reproductive capacity of adult mice was hindered,^18^ and while this was suggested to be caused by an age-related increase in DNA DSBs in surviving oocytes in mutant versus wild-type mice,^18^ it is more plausible this was attributed to reduced follicle endowment during embryonic development and bearing a heterozygous *Brca1*^+/Δ11^ mutation in every cell type. Furthermore, since heterozygous males were mated with heterozygous females, the reduced litter size likely culminated from lethality in homozygous embryos. Crucially, the ovarian reserve in aged mice or fertile lifespan were never assessed to determine whether or not ovarian ageing occurs in mutants. In an earlier study in fully grown oocytes, genetic knockdown by siRNA, or antibody-mediated protein depletion of BRCA1 in mouse oocytes was shown to impair meiotic spindle assembly and lead to misaligned chromosomes.^20^ Importantly though, complete genetic deletion culminating in complete loss of BRCA1 protein function, specifically in postnatal oocytes has never been studied across the fertile lifespan *in vivo*.

Here, we aimed to address this key knowledge gap and assess whether conditional loss of *Brca1* in oocytes impacts oocyte number, quality and fertility in mice with advancing maternal age. We also aimed to determine if AMH is an accurate marker of primordial follicle numbers using this *Brca1* conditional knockout model, as well as primary human ovarian tissues and serum from *BRCA1 or BRCA2* mutation carriers.

## Methods

### Animal ethics

All animal procedures and experiments were performed in accordance with the NHMRC Australian Code of Practice for the Care and Use of Animals and ARRIVE guidelines. The work was approved by the Monash Animal Research Platform Animal Ethics Committee (MARP-1 #30350, #18713).

### Generation of oocyte conditional *Brca1* knockout mice

Female *Brca1*^*fl/fl*^ mice with LoxP sites inserted into introns surrounding exon 11 in the *Brca1* locus maintained on an FVB/N background (RRID:MGI:5654755) were provided by Professor Jane Visvader, as described previously.^21^ Male transgenic mice that carried growth differentiation factor 9 (*Gdf9*) promoter-mediated Cre recombinase on a C57BL6/J background (RRID:MGI:5654755) were provided by Professor John Carroll as described previously,^22^^,^^23^ and then bred with FVB/*Brca1*^*fl/fl*^ females for 10 generations so that the breeder males were FVB/*Brca1*^*fl/fl*^*Gdf9*^*cre/+*^. To ensure *Brca1* gene deletion in oocytes only, FVB/*Brca1*^*fl/fl*^ female mice were then crossed with FVB/*Brca1*^*fl/fl*^*Gdf9*^*cre/+*^ males. Wild-type (WT: *Brca1*^*fl/fl*^*Gdf9*^*+/+*^) and oocyte conditional *Brca1* knockout (cKO: *Brca1*^*fl/fl*^*Gdf9*^*cre/+*^) female littermates were used in this study. All mice were housed in the Monash University Animal Research Laboratory under temperature-controlled and high barrier conditions, with free access to food and water, under a 12-h (h) light–dark cycle.

### Fertile lifespan assessment

Adult female mice (8 weeks of age; n = 9/genotype) were mated with proven C57BL6/J WT male studs at a 1:1 ratio, and kept for breeding until no litters had been produced for ≥2 months with two different male studs. The male stud was left in the cage throughout the duration of the study. If no litters were produced after 1 month, the male stud was replaced with another male. For analysis, time to first vaginal plug, first litter, age at last litter, total number of litters per female, total number of pups per female, litter size and gross observations of pup morphology, including offspring sex ratio and weights at PN5 and at PN20 at weaning were recorded. The offspring from each litter were housed with the dam and stud from birth until weaning at PN20, at which time offspring were humanely culled after being weighed.

### Superovulation and oocyte collection

For superovulation, female mice were administered an intraperitoneal injection of pregnant mare serum gonadotrophin (10 IU PMSG; #HOR-272, Prospec), followed 44–48 h later by human chorionic gonadotropin (10 IU hCG; #8713184 091796, MSD Animal Health). After 12–16 h, intact oocytes and cumulus cells were harvested from oviducts and mature metaphase-II (MII) stage oocytes were denuded by digestion in M2 medium (M#7167-50 mL, Sigma–Aldrich) containing 0.3% hyaluronidase (Sigma–Aldrich). The number of ovulated oocytes of each mouse was recorded and oocyte cytoplasm, first polar body, perivitelline space, zona pellucida, and meiotic spindle were assessed for quality.

### Oocyte *in vitro* maturation

For *in vitro* maturation studies, mice aged PN300 were administered with 10 IU of PMSG. After 44–48 h, the ovaries were dissected, and pre-ovulatory oocytes were harvested and placed into M2 medium. Intact germinal vesicle (GV) oocytes and cumulus cells were incubated in M16 medium (M72972-50 mL, Sigma–Aldrich). The proportions of oocytes that progressed to the MII phase (polar body extrusion), remained in metaphase I (MI), or that were dead or fragmented, were quantified after 12–14 h from n = 8 WT and n = 9 *Brca1* cKO mice across three independent experiments. Of note, n = 1 WT and n = 3 *Brca1* cKO PN300 mice did not respond to hormone stimulation and were hence excluded from IVM culture and analyses.

### Oocyte immunofluorescence staining

Oocytes were fixed in 4% paraformaldehyde (PFA) and permeabilized in 2% Triton X-100 solution for 30 min (mins), washed in blocking buffer (1% BSA/phosphate-buffered saline [PBS] + 1:5000 Tween-20), and then blocked at room temperature for 1 h. Subsequently, oocytes were stained with 1:5000 Hoechst 33342 (Thermo Scientific, 62249) for 10 min with 1:100 anti–β-tubulin (Sigma–Aldrich, T4026) for 1 h. A Leica SP8 Invert microscope was used for imaging and FIJI software was applied to process and analyse the images.

### Animal tissue collection

At postnatal day (PN) 5, 20, 50, 200 and 300, female WT and cKO mice were humanely killed by isoflurane inhalation, followed by cardiac puncture to collect peripheral blood. Serum was separated by centrifugation, then stored at −80 °C. Mice were weighed at end point and one ovary harvested, snap frozen, then stored at −80 °C, and the other fixed in 10% (vol/vol) neutral buffered formalin solution for 24 h before being paraffin-embedded.

### Assessment of puberty onset in mice

Puberty checks were performed daily from PN18 onwards by experienced investigators until the first day of vaginal opening was recorded, signifying the onset of puberty as described previously.^24^

### Mouse ovarian follicle counts

To estimate ovarian follicle numbers, one paraffin-embedded ovary per animal was exhaustively serially sectioned at 5 μm and every ninth tissue section collected and stained with periodic acid-Schiff and haematoxylin. Whole tissue images were captured on the Aperio Digital Pathology Slide Scanner (Leica Biosystems) at × 20 objective, then visualised by a blinded assessor using Aperio ImageScope software (Leica Biosystems). Total number of healthy primordial, primary, secondary and antral follicles, as well as atretic secondary and antral follicles were quantified in every ninth section of each ovary using a similar strategy as previously described.^25^^,^^26^ Follicles were counted if the oocyte nucleus was present. Total follicle numbers were obtained by multiplying the raw counts of follicles sampled by nine, to correct for sections not counted.

### Mouse ovarian tissue immunofluorescence staining

To assess levels of DNA damage in primordial follicles, immunofluorescence staining for γH2AX – a marker of DNA DSBs – was performed as previously described,^27^ with minor adaptations. Briefly, slides (4 sections per animal with a minimum interval of 40 μm between sections; n = 5 animals/genotype) were deparaffinized in histolene then rehydrated in a series of graded ethanols. Next, antigen retrieval was performed by microwaving sections in pre-warmed sodium citrate buffer (0.01M, pH 6) for 8 min, then slides were left to cool in the buffer for 30 min. Sections were blocked with 10% donkey serum in 3% BSA for 30 min. Sections were then incubated overnight at 4 °C in 5% donkey serum in 3% BSA combined with primary antibodies against γH2AX (1:500; Cell Signalling Technology, 9718) and c-Kit (1:500; R&D Systems, AF1356). A biological positive control was included in every run, with known DNA damage resulting from treatment with the chemotherapy 4-hydroperoxycyclophosphamide (4-HC). For negative controls, γH2AX antibodies were omitted. Sections were incubated with the secondary antibodies donkey anti-goat Alexa Fluor 488™ (1:500; Invitrogen, A11055) and donkey anti-rabbit Alexa Fluor 568™ (1:500; Invitrogen, A10042) for 1 h at room temperature. Nuclear staining was performed by incubating sections in Hoechst 33258 (1:2000; ThermoFisher Scientific, H3569) for 10 min at room temperature. Slides were cover-slipped with FluorSave reagent (Sigma–Aldrich, #345789) and cured for 24 h at room temperature. Sections were imaged using a Nikon C1 Invert confocal microscope (Nikon Corp., Tokyo, Japan; Monash Micro Imaging Platform, Monash University, Clayton VIC) with a × 40 oil immersion objective and 405/488/561 laser lines. Images were processed in FIJI software (NIH, Schindelin 2012).

Primordial follicles with bright, c-Kit-positive staining (an oocyte marker) and a complete, distinct, DAPI-positive margin of the oocyte nucleus were selected for analysis. Follicles were classed as positive if ≥ 2 distinct, bright γH2AX foci were present within the oocyte nucleus. Follicles with only 1 focus, or diffuse nuclear staining with no foci, were classed as negative.

### Mouse anti-Müllerian hormone enzyme linked immunosorbent assay

Mouse serum AMH concentrations were determined in duplicate using the mouse AMH ELISA (#AL-113, Ansh Labs) according to the manufacturer's instructions, and absorbance measured using the ClarioStar microplate reader (BMG Labtech).

### Human ethics

Ethics approval was granted by University of Melbourne to access the What Happens after Menopause? (WHAM) cohort biobank (HREC12PMCC24-12/90).

### Human ovarian tissue and serum collection

WHAM is a prospective, controlled study of premenopausal women with a *BRCA1/2* mutation, aged between 18 and 45 years and undergoing risk-reducing bilateral salpingo-oophorectomy (RRBSO).^28^ None had ovarian cancer diagnosed at the time of surgery and all had serum collected prior to oophorectomy for measurement of AMH. Ovarian cortical tissue biopsies were obtained by accessing the WHAM cohort biobank. Exclusion criteria for this study were previous chemotherapy treatment likely to impact on ovarian reserve, personal history of ovarian cancer, use of hormonal contraception at the time of blood draw or oophorectomy, ovarian cancer diagnosed at oophorectomy, or polycystic ovary syndrome.^29^

### Human ovarian tissue immunohistochemistry

Forty slides consisting of one ovarian tissue section (5 μm) per ovary (*BRCA1* mutation carriers n = 8; *BRCA2* mutation carriers n = 10) were systematically selected and then deparaffinized in histolene, before being rehydrated in a graded series of ethanols. Antigen retrieval was performed with sodium citrate (pH 6). Endogenous peroxidases were quenched, and non-specific binding of antibodies was blocked with 10% normal goat serum in 0.1 M Tris, 150 mM NaCl, and 0.1% v/v Tween 20 buffer (TNT). Tissues were incubated with primary DEAD-Box Helicase 4 (DDX4) antibody (Abcam #27591) 1:2000 in TN overnight at 4 °C, while a negative isotype control of non-immunized mouse IgG (Vector Laboratories) was included on one slide per run. Tissue sections were incubated with biotinylated goat antibody against mouse IgG (1:500; Vector Laboratories), followed by avidin–biotin peroxidase complex (Vector Laboratories), then 3,3′-diaminobenzidine and sections were counterstained with haematoxylin. Whole tissue section images were captured on the Aperio Digital Pathology Slide Scanner (Leica Biosystems) at × 20 objective, then visualised by a blinded assessor using Aperio ImageScope software (Leica Biosystems) and counted as described below.

### Human ovarian follicle counts

Direct follicle counts were performed at an interval of every 4th section (5 μm) of the human ovarian cortical tissue samples across forty sections and classified as either primordial or growing follicles by two experienced, blinded assessors according to McLaughlin et al.^30^ Follicles were counted if the nucleus was present. The interval of 4 was determined by measuring the average diameter of oocyte nuclei present within these sections. A total of 15 oocyte nuclei were measured from follicles at different stages of growth, across ovarian samples from three different individuals. The average nuclei diameter was then determined to be approximately 16.58 μm. As the ovary was serially sectioned at 5 μm, counting every 4th section meant conducting direct follicle counts for each 20 μm of the ovary, ensuring that follicles were not double counted. Cortical ovarian tissue section area was quantified using Aperio ImageScope software (Leica Biosystems). The follicle density per area (μm^2^) was then calculated using the following formula (follicle count/area of ovarian tissue section) and repeated for all analysed sections and summed together as previously established.^31^^,^^32^ The follicle density per volume (μm^3^) was calculated using the following formula (follicles/(area x thickness of sample)). The abnormal follicle rate (%) was calculated using the following formula (abnormal follicle count/[abnormal follicles + healthy follicles] x 100).

### Human anti-Müllerian hormone enzyme linked immunosorbent assay

Human serum AMH concentrations were determined in duplicate using the human picoAMH ELISA (#AL-124, Ansh Labs) according to the manufacturer's instructions and absorbance measured using the ClarioStar microplate reader (BMG Labtech).

### Statistics

Data are presented as mean ± SEM and statistical analysis was performed using GraphPad Prism Software. Normality was tested using a Shapiro–Wilk normality test. Normally distributed (parametric) data were analysed by Student's t-test to compare two groups (WT versus *Brca1* cKO) within each age group and respective parameter assessed (e.g. follicle number).

Similarly, non-parametric data were analysed using a Mann–Whitney test to compare two groups. For all correlation plots, Spearman's rank correlation test was performed, which assumes that the data are paired observations with a non-parametric distribution and a monotonic relationship (independent variable: follicle number or density, dependent variable: AMH concentration; note: age was not included as a variable due to the small sample size). Proportions of oocyte *in vitro* maturation were analysed using Fisher's exact test. Differences were considered significant when p < 0.05. For animal studies, we performed power calculations to determine animal numbers required to be sufficient for statistical analysis at 80% power. These were calculated to detect a 25% change in litter size (at least n = 7/genotype required) and a 20% change in ovarian follicle number (n = 4/age/genotype required) with a p-value <0.05.

### Role of funders

The Funders of this study did not have any role in study design, data collection, data analyses, interpretation, or writing of report.

## Results

### Conditional loss of *Brca1* in oocytes leads to reduced litter size, but normal fertile lifespan in female mice

**Fig. 1:**
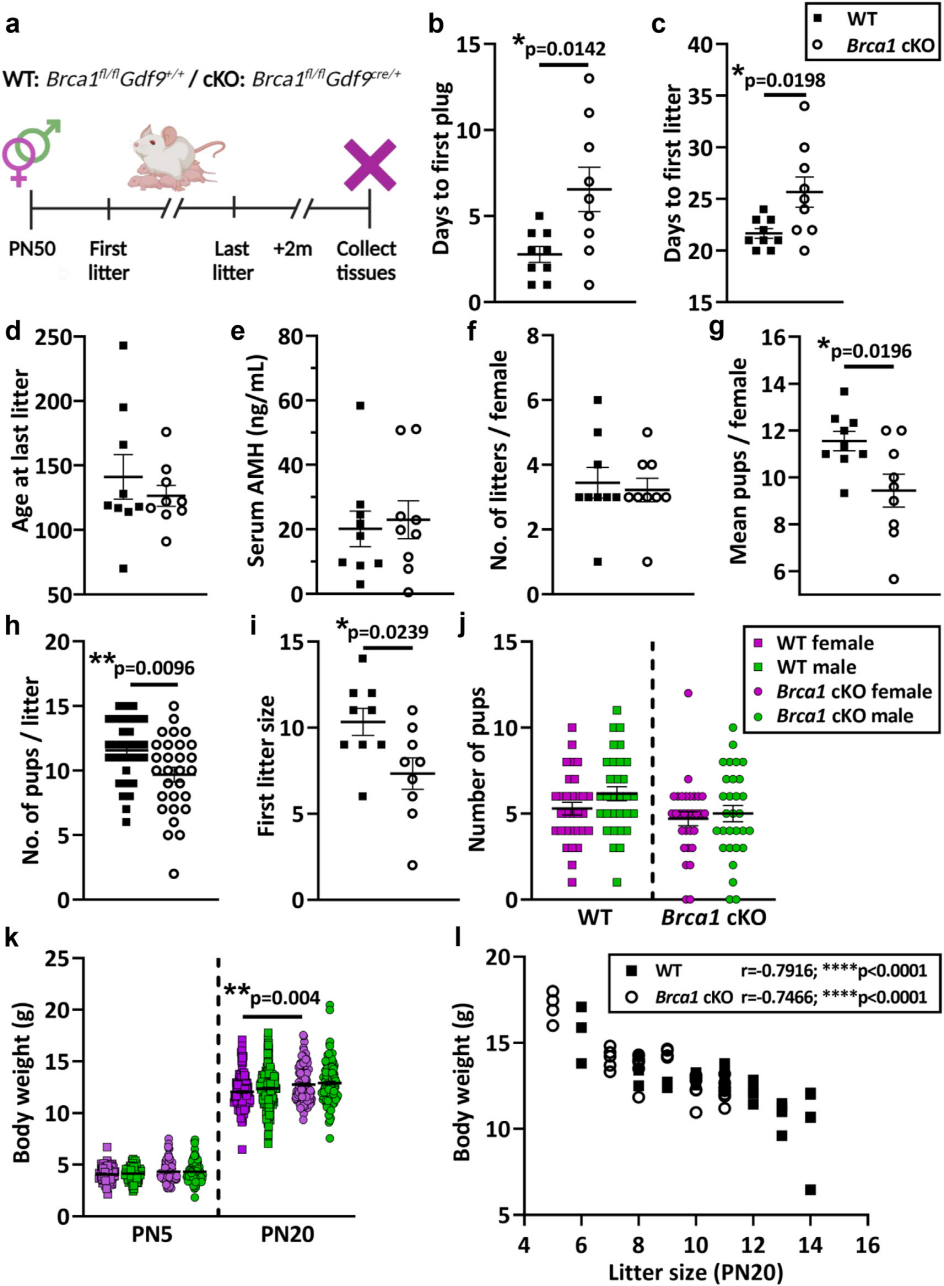
**(a)** Schematic representation of the fertile lifespan breeding trial of wild-type (WT: Brca1^fl/fl^Gdf9^+/+^) and oocyte conditional *Brca1* knockout (cKO: Brca1^fl/fl^Gdf9^cre/+^) female mice (n = 9/genotype) mated with proven C57BL6/J WT male studs. Females were kept for breeding for their entire fertile lifespan, until no litters had been produced for ≥2 months. **(b)** Time to first vaginal plug, **(c)** time to first litter and **(d)** age at last litter were recorded. **(e)** Maternal serum AMH concentrations (ng/mL) were measured at necropsy. **(f)** Total number of litters per female, **(g)** average number of pups per female across all litters **(h)** size of each litter and **(i)** first litter size were recorded. Data are mean ± SEM; unpaired t-test; ∗p < 0.05, ∗∗p < 0.01. **(j)** Total number of male and female offspring and **(k)** offspring weights per sex at postnatal day (PN)5 and PN20 were recorded. **(l)** Offspring weight at PN20 was adjusted for litter size for each genotype by Spearman's rank correlation test. Data are mean ± SEM; Kruskal–Wallis test; ∗∗p < 0.01, ∗∗∗∗p < 0.0001.

To comprehensively evaluate the contribution of BRCA1 to oocyte maintenance and survival across the fertile lifespan, we generated a conditional knockout model whereby the *Brca1* gene was deleted only in oocytes. To do this, *Brca1*^*loxP/loxP*^ mice^21^ were crossed with transgenic mice expressing growth differentiation factor 9 (*Gdf9*) promoter-mediated Cre recombinase^22^ (Supplementary Fig. S1). In *Gdf9-cre* mice, Cre is specifically expressed in oocytes of follicles at the primordial stage and beyond^23^ and the knockout in this model was validated (Supplementary Figs. S2 and S3). To assess the fertile lifespan of mice with conditional loss of *Brca1* in oocytes, wild-type (WT: *Brca1*^*fl/fl*^*Gdf9*^*+/+*^) and oocyte conditional *Brca1* knockout (cKO: *Brca1*^*fl/fl*^*Gdf9*^*cre/+*^) female mice (n = 9/genotype) were mated with wild-type C57BL6/J males of proven fertility for their entire fertile lifespan, until no litters had been produced for ≥2 months (Fig. 1a). In *Brca1* cKO animals, the time to first vaginal plug was significantly increased compared to WT (WT: 2.8 days ±0.46, *Brca1* cKO: 6.6 days ±1.3, p = 0.0142, unpaired t-test) (Fig. 1b). Time to first litter was also significantly increased in *Brca1* cKO animals versus WT (WT: 21.7 days ±0.47, *Brca1* cKO 25.7 days ±1.4, p = 0.0198, unpaired t-test) (Fig. 1c). At the conclusion of the breeding trial, there were no differences in the age at last litter (Fig. 1d), or serum AMH concentrations between genotypes (Fig. 1e). The total number of litters per female was similar between genotypes (Fig. 1f), though the average number of pups per female across all litters was significantly reduced in *Brca1* cKO animals versus WT (WT: 11.6 pups ± 0.4, *Brca1* cKO: 9.4 pups ± 0.7, p = 0.0196, unpaired t-test) (Fig. 1g). Similarly, average litter size was significantly reduced in *Brca1* cKO animals versus WT, across all litters (WT: 11.6 pups ± 0.4, *Brca1* cKO: 9.7 pups ± 0.5, p = 0.0096, unpaired t-test) (Fig. 1h). Analysis from the first litter of each female revealed that litter size was significantly reduced in *Brca1* cKO mothers compared to WT (WT: 10.3 pups ± 0.8, *Brca1* cKO: 7.3 ± 0.9, p = 0.0239, unpaired t-test) (Fig. 1i). There were no sex differences (number of males versus females) in the offspring between genotypes across all litters (Fig. 1j), or differences in offspring weights between genotypes at PN5 (Fig. 1j). However, by PN20, female offspring from *Brca1* cKO mothers were significantly heavier compared to females from WT mothers (WT: 12.1 g ± 0.1, cKO: 12.8 g ± 0.1, p = 0.0040, Kruskal–Wallis test) (Fig. 1k). Although, correlation analysis was performed between the number of pups per litter and body weight, demonstrating that this weight difference was attributed to a litter size effect (WT: r = −0.7916, p < 0.0001; *Brca1* cKO: r = −0.7466, p < 0.0001, Spearman correlation) (Fig. 1l). Notably, the delays in the time to litter and reduction in litter size were only significantly altered in the first litter of *Brca1* cKO mothers compared to WT (Supplementary Fig. S4), although this was likely to be attributed to the reduced statistical power with the increasing number of litters, as few females had more than three litters.

### Oocyte maturation is impaired with advanced maternal age in *Brca1* cKO mice

**Fig. 2:**
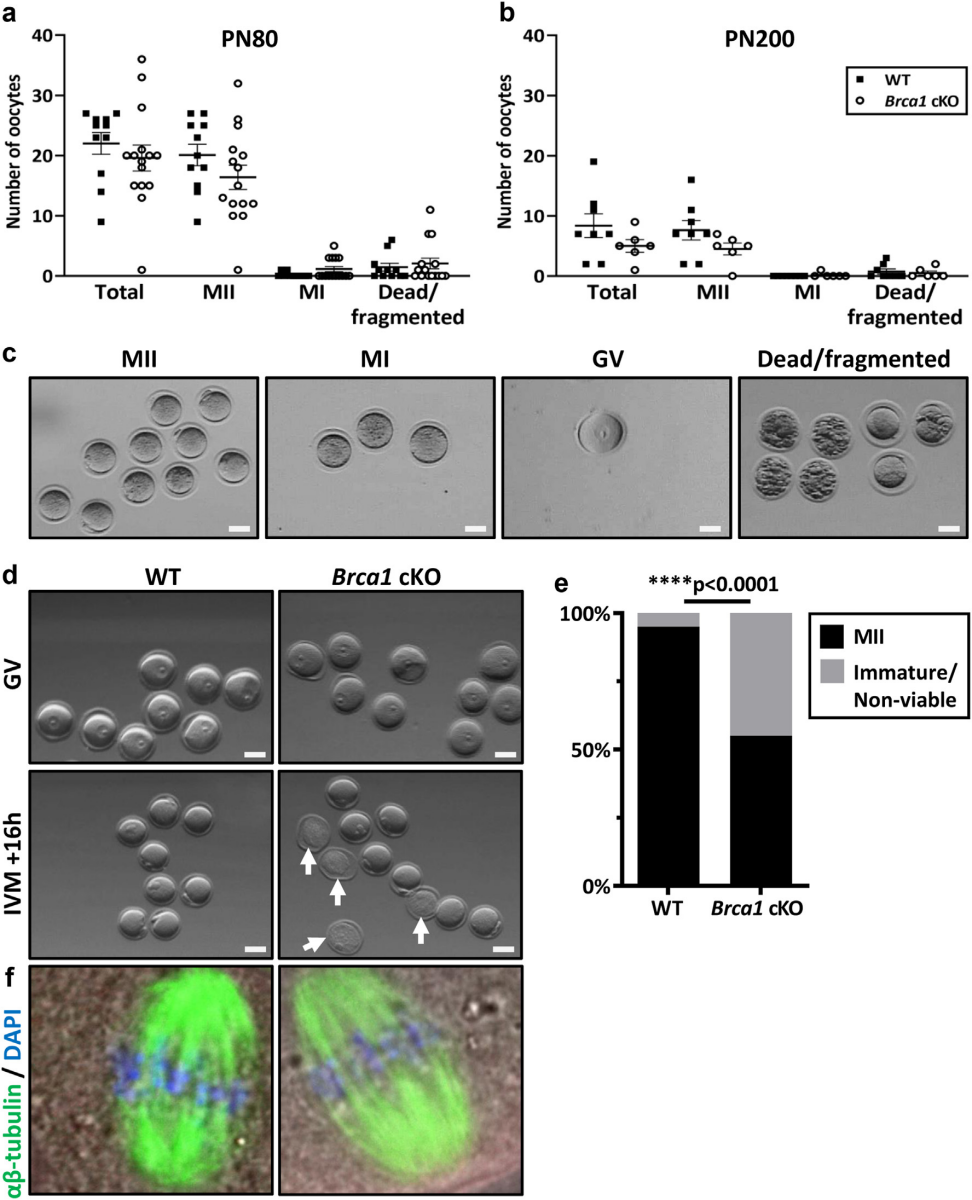
**(a)** Numbers of ovulated oocytes harvested following exogenous hormonal stimulation from WT and *Brca1* cKO mice at PN80 (WT n = 11; *Brca1* cKO n = 15) and **(b)** PN200 (WT n = 8; *Brca1* cKO n = 6). Data are presented as mean ± SEM; t-test**. (c)** Representative images of intact mature MII, immature MI, immature germinal vesicle (GV) or fragmented/dead oocytes obtained from control WT and *Brca1* cKO animals at PN80; bar = 50 μm. **(d)** The top panel shows representative images of GV oocytes harvested from PN300 WT or *Brca1* cKO animals before *in vitro* maturation (IVM) culture was performed for 16 h; bar = 50 μm. The lower panel shows representative images of either mature MII oocytes, or a combination of MII or immature or non-viable oocytes (arrows) following IVM from WT or *Brca1* cKO PN300 mice; bar = 50 μm. **(e)** The maturation rate (%) from GV to MII oocytes for each genotype is depicted in black bars, and proportion (%) of immature or non-viable oocytes are depicted in grey bars. Proportions are calculated from oocytes derived from n = 8 WT and n = 9 *Brca1* cKO mice across three independent experiments; Fisher's exact test, ∗∗∗∗p < 0.0001. **(f)** PN300 MII oocytes harvested following IVM were immunostained with αβ-tubulin (green) to label the meiotic spindle, and DAPI (blue) to label the DNA on the metaphase plate.

To determine whether reduced litter size in *Brca1* cKO female mice is attributed to a reduction in oocyte quality, animals were stimulated with exogenous hormones and oocytes harvested at PN80 (peak fertility) and PN200 (advancing maternal age). At PN80, there were no differences in the numbers (Fig. 2a) or proportions (Supplementary Fig. S5a) of total, mature (MII), immature (MI), or dead or fragmented oocytes between genotypes. Similar results were observed at PN200 (Fig. 2b; Supplementary Fig. S5b). Representative images of these oocyte classifications are depicted (Fig. 2c). A critical indicator of oocyte quality is the ability to resume meiosis and progress to the metaphase II stage (MII), a process referred to as meiotic maturation. Therefore, we next performed *in vitro* maturation assays at PN300 (advanced reproductive age) to provide insight into the impact of BRCA1 function on oocyte development at advanced age (Fig. 2d). Strikingly though, *Brca1* loss in oocytes significantly impaired maturation to MII by 45% compared to WT (Fig. 2e), demonstrating defective oocyte maturation. However, chromatin structure was normal in all oocytes that did mature from each genotype (Fig. 2f).

### Conditional loss of *Brca1* in oocytes leads to reduced ovarian reserve with advanced maternal age

**Fig. 3:**
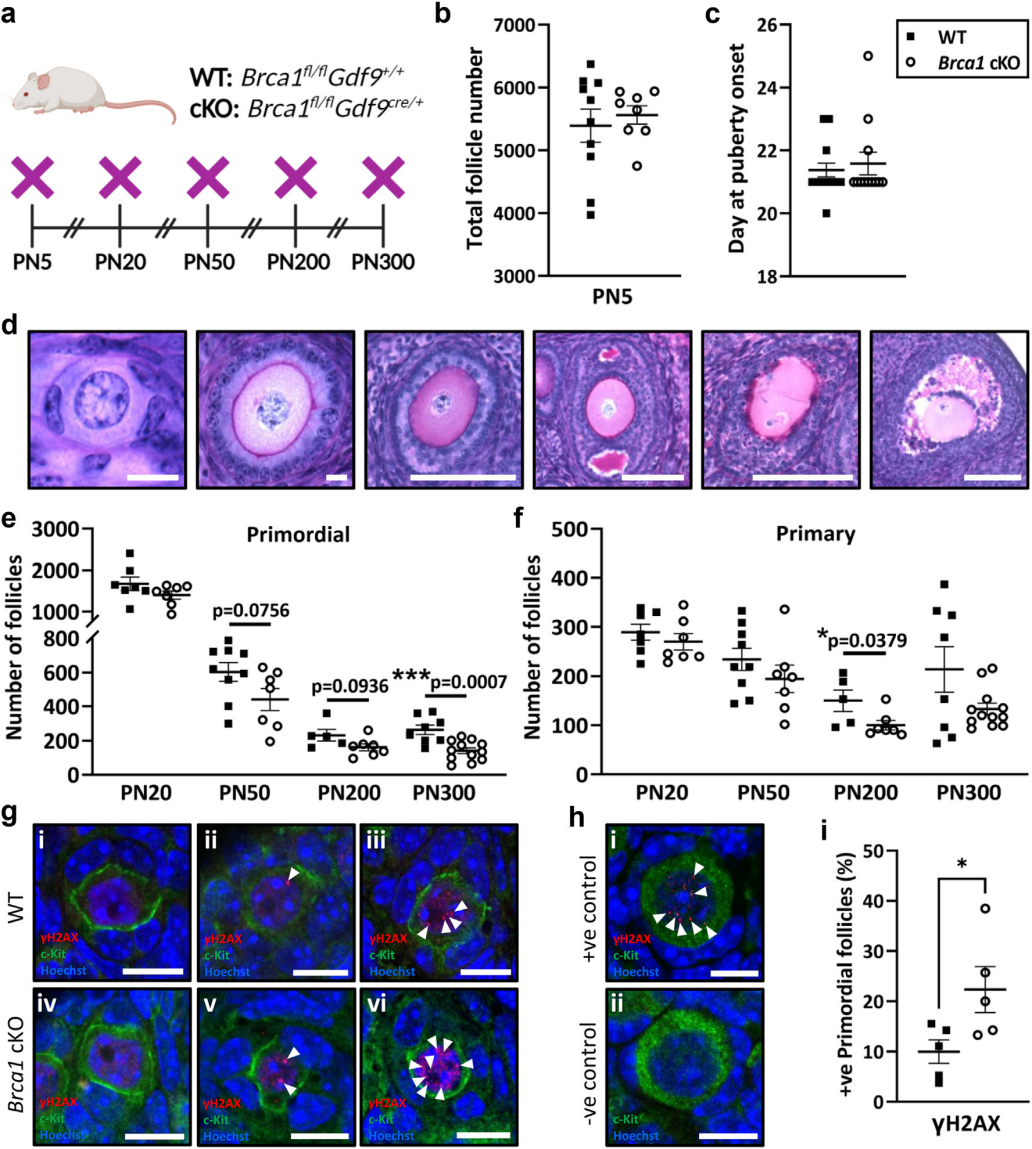
**(a)** Schematic representation of animals used for ovarian follicle counts from WT and *Brca1* cKO female mice aged PN5, PN20, PN50, PN200 and PN300 (n = 6–10/age/genotype). **(b)** Ovarian follicle endowment was assessed by quantifying total follicles in ovaries from mice aged PN5 (WT n = 10; *Brca1* cKO n = 8). **(c)** Age at vaginal opening was assessed to determine puberty onset (WT n = 16; *Brca1* cKO n = 12). **(d)** Representative photomicrographs of periodic acid Schiff (PAS) stained ovarian follicles: **i.** primordial; bar = 10 μm, **ii.** primary; bar = 10 μm, **iii.** secondary; bar = 100 μm, **iv.** antral; bar = 200 μm, **v.** atretic secondary; bar = 100 μm and **vi.** atretic antral; bar = 200 μm. **(e)** Primordial and **(f)** primary follicles were quantified (WT black squares: PN20 n = 7, PN50 n = 9, PN200 n = 5, PN300 n = 8; *Brca1* cKO open circles: PN20 n = 7, PN50 n = 7, PN200 n = 7, PN300 n = 12). **(g)** Ovarian tissue sections were immunostained with c-Kit to label oocytes and γH2AX to detect the accumulation of endogenous DNA damage in WT and *Brca1* cKO primordial follicle oocytes at age PN50. Representative images of γH2AX staining in primordial follicles are shown; specifically, **(i-ii)** negative and **(iii)** positive follicles from WT animals, as well as **(iv)** negative and **(v-vi)** positive follicles from *Brca1* cKO animals; arrowheads = γH2AX foci; red = γH2AX, green = c-Kit; blue = Hoechst; bars = 10 μm. **(h)** Representative images of primordial follicles from **(i)** positive and **(ii)** negative controls are shown. **(i)** The proportion of γH2AX-positive primordial follicles were quantified in ovaries from WT and *Brca1* cKO animals. A total n = 241 primordial follicles were analysed across n = 4 sections/ovary from n = 5 mice/genotype. Data are presented as mean ± SEM; unpaired t-test or Mann–Whitney test; ∗p < 0.05, ∗∗∗p < 0.001.

To determine whether reductions in oocyte number of *Brca1* cKO animals contributed to reduced litter size, ovarian follicles of each class were enumerated in whole ovaries from WT and *Brca1* cKO females throughout the lifespan at PN5, PN20, PN50, PN200 and PN300 (Fig. 3a). Females of each genotype were endowed with equal numbers of total ovarian follicles at PN5, ranging between an average of 5392–5562 total follicles per ovary (Fig. 3b). The time to onset of puberty was unaltered in females of each genotype, which occurred between an average of 21–22 days (Fig. 3c). Ovarian follicles were quantified and classified morphologically as either primordial (Fig. 3d i), primary (Fig. 3d ii), secondary (Fig. 3d iii) or antral follicles (Fig. 3d iv). Additionally, follicle heath was assessed histologically and atretic (i.e. dying) secondary (Fig. 3d v) and antral follicles (Fig. 3d vi) were also quantified. Primordial follicle numbers were similar between genotypes at PN20, but trending towards a reduction in *Brca1* cKO at PN50 and PN200 and significantly reduced by 47% in *Brca1* cKO animals versus WT by PN300 (WT: 264 ± 27, *Brca1* cKO: 141 ± 17, p = 0.0007, unpaired t-test) (Fig. 3e). Primary follicle numbers were similar between genotypes at PN20, PN50 and PN300, but significantly reduced by 33% in *Brca1* cKO animals compared to WT at PN200 (WT: 150 ± 22, *Brca1* cKO: 100 ± 10, p = 0.0435, Mann–Whitney test) (Fig. 3f).

To assess endogenous levels of DNA damage accumulation in primordial follicle oocytes from WT and *Brca1* cKO animals at PN50, γH2AX – a marker of DNA DSBs – was immunolocalised (Fig. 3g and h). This age was chosen as primordial follicles are relatively abundant, and trends towards decreased primordial follicles began to be observed in *Brca1* cKO animals (Fig. 3e). Interestingly, a significant 2.2-fold increase in the proportion of γH2AX-positive primordial follicles was observed in *Brca1* cKO animals versus WT (WT: 10.0 ± 2.4%, *Brca1* cKO: 22.4 ± 4.6%, p = 0.0438, unpaired t-test), indicating that these follicles are sustaining higher levels of DNA damage endogenously (Fig. 3h).

**Fig. 4:**
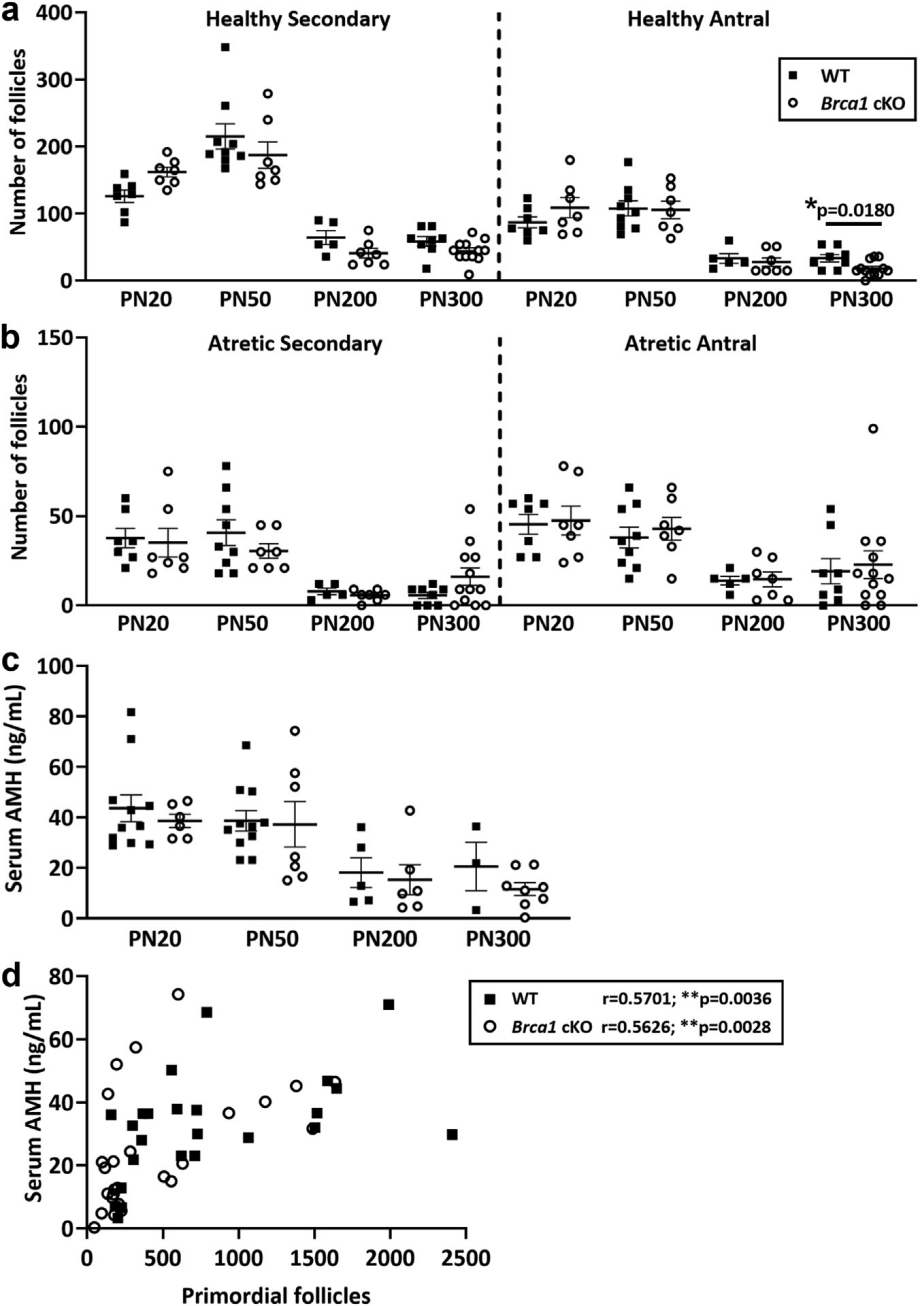
**(a)** Healthy secondary and antral and **(b)** atretic secondary and antral follicles were quantified in ovaries from animals at ages PN20, PN50, PN200 and PN300 based on follicle classifications represented in Fig. 3d (WT black squares: PN20 n = 7, PN50 n = 9, PN200 n = 5, PN300 n = 8; *Brca1* cKO open circles: PN20 n = 7, PN50 n = 7, PN200 n = 7, PN300 n = 12). **(c)** Serum AMH concentrations (ng/mL) from female WT and *Brca1* cKO animals at ages PN20, PN50, PN200 and PN300 were measured (WT: PN20 n = 11, PN50 n = 11, PN200 n = 5, PN300 n = 3; *Brca1* cKO: PN20 n = 6, PN50 n = 7, PN200 n = 6, PN300 n = 8). **(d)** Matched sample serum AMH concentrations (ng/mL) (dependant variable) were measured and correlated by Spearman's rank correlation test with total primordial follicle number (independent variable) in WT (n = 42) and *Brca1* cKO mice (n = 50) across all ages. Data are presented as mean ± SEM; unpaired t-test; ∗p < 0.05, ∗∗∗p < 0.001.

The numbers of healthy growing (Fig. 4a) and atretic growing follicles (Fig. 4b) were similar between genotypes at each age, with the exception of healthy antral follicles, which were significantly reduced by 45% in *Brca1* cKO animals compared to WT at PN300 (WT: 33 ± 5, *Brca1* cKO: 18 ± 3, p = 0.0180, unpaired t-test) (Fig. 4b). Overall, these data indicate that conditional loss of *Brca1* in oocytes leads to an age-dependent reduction in oocyte number.

### Serum AMH concentrations are not predictive of reduced primordial follicle number in *Brca1* cKO mice with advancing maternal age

Circulating AMH concentrations are widely used as a surrogate marker of the ovarian reserve in clinical practice. Serum AMH concentrations were measured in female mice at PN20, PN50, PN200 and PN300, with no differences in levels between genotypes at all ages (Fig. 4c). Although a statistically significant correlation between AMH levels and primordial follicle number was observed, this correlation was only moderate in both WT (r = 0.5701, p = 0.0036, Spearman correlation) and *Brca1* cKO animals across all ages (r = 0.5626, p = 0.0028, Spearman correlation), as indicated by low r values (Fig. 4d). Notably, AMH was not predictive of reduced primordial follicle numbers detected between genotypes.

### Serum AMH concentrations do not correlate with primordial follicle numbers in human ovarian tissues from *BRCA1* or *BRCA2* mutation carriers

**Fig. 5:**
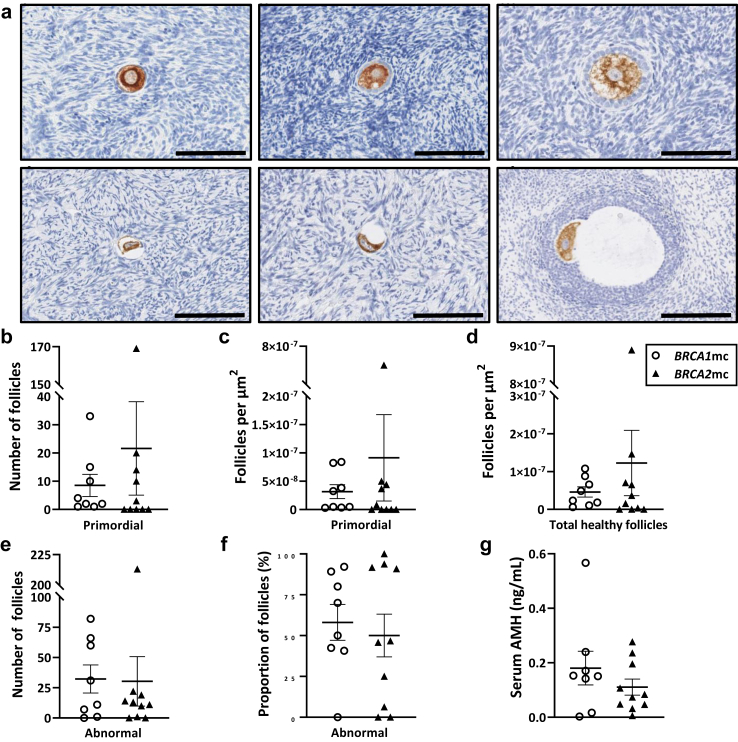
**(a)** Human cortical ovarian tissues from *BRCA1* (n = 8) and *BRCA2* (n = 10) mutation carriers (mc) were immunohistochemically stained for DDX4 as a marker of oocytes. Photomicrographs are representative of the classifications used to identify **i.** primordial follicles; bar = 100 μm, **ii.** primary follicles; bar = 100 μm, **iii.** secondary follicles; bar = 100 μm **iv-v.** abnormal follicles; bar = 100 μm **vi.** abnormal follicles; bar = 200 μm. **(b)** Total primordial follicle number, **(c)** primordial follicle density per tissue area (μm^2^) and **(d)** total healthy follicle density per tissue area (μm^2^) were enumerated. **(e)** Total abnormal follicle number and **(f)** abnormal follicle rate (%) were quantified. **(g)** Serum AMH levels were quantified by ELISA. Data are mean ± SEM; unpaired t-test.

To determine the relevance of AMH as a marker of primordial follicles in the human ovary, we correlated primordial follicle counts with matched serum AMH concentrations. To do this, DDX4-positive follicles were staged (Fig. 5a) and enumerated from human cortical ovarian tissue sections from premenopausal women with *BRCA1* or *BRCA2* mutations who underwent RRBSO. AMH concentrations were measured in serum from blood drawn prior to RRBSO. There were no differences in the numbers (Fig. 5b), density by area (Fig. 5c) or density by volume of primordial follicles (Supplementary Fig. S6a) between *BRCA1* or *BRCA2* mutation carriers. Healthy ovarian follicle density by area (Fig. 5d) and by volume (Supplementary Fig. S6b) were similar for all follicle classes between *BRCA1* or *BRCA2* mutation carriers. There were no differences in the numbers (Fig. 5e) or density of abnormal or atretic follicles (Fig. 5f) between *BRCA1* or *BRCA2* mutation carriers. Serum AMH concentrations were also similar between *BRCA1* or *BRCA2* mutation carriers (Fig. 5g). Spearman rank correlation analysis demonstrated no correlation between primordial follicle number and AMH concentration in *BRCA1* (r = 0.3615, p = 0.3776, Spearman correlation) or *BRCA2* (r = 0.6013, p = 0.0723, Spearman correlation) mutation carriers (Supplementary Fig. S6c). This was similar for correlations of AMH levels with primordial follicle density by area or volume between *BRCA1* (r = 0.4286, p = 0.2992, Spearman correlation) or *BRCA2* (r = 0.5754, p = 0.0880, Spearman correlation) mutation carriers (Supplementary Fig. S6d and e). This is unsurprising, as AMH is produced by the granulosa cells of growing ovarian follicles, not primordial follicles. Interestingly though, no correlation was observed between growing follicle number and AMH concentration in *BRCA1* (r = 0.6131, p = 0.1060, Spearman correlation)) or *BRCA2* (r = 0.5049, p = 0.1367, Spearman correlation) mutation carriers (Supplementary Fig. S6f), or total follicle number between *BRCA1* (r = 0.4192, p = 0.3034, Spearman correlation) or *BRCA2* (r = 0.5170, p = 0.1295, Spearman correlation) mutation carriers (Supplementary Fig. S6g), indicating that AMH may not be a reliable marker of the ovarian reserve.

## Discussion

In the present study, we comprehensively assessed the ovarian reserve of mice with a conditional knockout of *Brca1* in oocytes across the lifespan, ranging from PN5 to PN300, using females derived from multiple litters for every parameter assessed. Our data in mice demonstrate that conditional loss of *Brca1* in oocytes leads to reduced litter size, and a decline in the ovarian reserve and impaired maturation potential of oocytes with advanced maternal age. In addition, in a small cohort, we correlated primordial follicle counts and density in human ovarian cortical tissues with serum AMH concentrations from *BRCA1/2* gene mutation carriers. Our data show no differences in follicle numbers, follicle density, or serum AMH concentrations between *BRCA1* versus *BRCA2* gene mutation carriers. Furthermore, our human data reveal no correlation between circulating AMH concentrations and the ovarian reserve in this small cohort.

The initial hypothesis that *BRCA1/2* mutation carriers have a reduced ovarian reserve was derived from observations of poor response to hormonal stimulation during IVF treatment compared to non-mutation carriers.^33^^,^^34^ A larger study that has been performed since was not able to confirm those findings^35^ and another subsequent study actually showed greater numbers of mature oocytes retrieved from *BRCA* gene mutation carriers compared to age-matched women from the general population.^36^ These conflicting clinical reports did not differentiate between *BRCA1* and *BRCA2* mutations, and it is possible that additional factors may have also influenced responsiveness beyond ovarian reserve, including the gonadotropin dose, protocol, and degree of suppression. In preclinical models, a previous study by Titus et al. also showed fewer mature oocytes retrieved in *Brca1*^+/Δ11^ mutants versus WT in response to ovarian stimulation, although that mouse model was endowed with fewer ovarian follicles. In contrast, our model assessed the functional consequences of loss of BRCA1 protein function in postnatal oocytes *in vivo*. While we did not find an altered number of mature oocytes retrieved from *Brca1* cKO mice at PN80 or PN200 in response to hormone stimulation, *in vitro* oocyte maturation was dramatically impaired by 45% in *Brca1* cKO oocytes by PN300. This suggests an age-related decline in oocyte quality in response to loss of function of BRCA1 protein.

Since ovarian reserve exhaustion dictates the onset of menopause, age at menopause has been investigated in cross sectional studies of human *BRCA* gene mutation carriers. In some earlier reports, no differences in age at menopause were found in *BRCA* mutation carriers versus non-mutation carrier relatives.^37^^,^^38^ In those cases, determining the age at menopause in *BRCA* mutations carriers was confounded by the risk of cancer treatments causing POI, as well as the use of chemoprevention strategies, such as tamoxifen or, RRBSO.^38^ Notably though, more recent largescale GWAS performed in over 45,000 women specifically implicate loss of function *BRCA1* and *BRCA2* variants in earlier age of menopause versus non-mutation carriers.^7^ While mice do not undergo menopause, reproductive senescence does occur. In the present study, *Brca1* cKO mice had a significantly reduced litter size across their fertile lifespan and significantly reduced primordial follicles remained in *Brca1* cKO mice versus WT by PN300. These findings support that women with a *BRCA1* mutation may experience accelerated fertility decline with advancing maternal age, as well as early onset menopause.

Unfortunately, direct changes to the primordial follicle pool are invariably difficult to quantify in women, due to a lack of available biomarkers or analytical techniques. There are no established direct markers for quantifying the ovarian reserve in women, and the gold standard remains arduous enumeration of primordial follicles in entire ovaries or cortical sections.^39^ Assessing the total number of primordial follicles requires whole ovaries, but primordial follicle density can be calculated from cortical tissue sections, and this approach has been validated in fertile women against whole ovary data.^31^ Only two small studies have measured primordial follicle density in *BRCA1/2* mutation carriers with conflicting results. One small study of ovarian sections from 18 *BRCA1*/2 mutation carriers compared to ovarian sections from 12 organ donation cadavers, showed primordial follicle density was significantly decreased in mutation carriers and increased DNA DSBs were evident in *BRCA* mutations carriers, indicating defective DNA repair.^40^ Another small study reported decreased primordial follicle density from 15 *BRCA1*/2 mutation carriers versus non-mutation carriers undergoing RRBSO.^41^ However, in these studies, results were not broken down by gene mutation type, and *BRCA* mutation carriers were older than non-mutation carriers.^40^^,^^41^ In the present study, we did not report any differences in follicle number or density between *BRCA1* and *BRCA2* mutation carriers. Notably though, this is a small cohort, and a further limitation of the present study is a lack of a proper control non-mutation carrier group, indicating that broader population studies should be conducted.

Serum anti-Müllerian hormone (AMH) concentrations are widely used clinically as a surrogate marker of the ovarian reserve.^6^ However, previous studies that quantified AMH levels in *BRCA1/2* gene mutation carriers report contradictory results. One study reported no differences between 124 premenopausal women with *BRCA1/2* mutations and 131 age-matched non-mutation carriers.^42^ Reports from some larger cohorts found serum AMH concentrations may differ based on gene mutation, with lower levels detected in aged-matched *BRCA1* versus *BRCA2* mutation carriers.^43^, ^44^, ^45^ But importantly, AMH is secreted from granulosa cells of growing follicles,^3^^,^^46^^,^^47^ not primordial follicles. Serum AMH levels peak between the age of 20–25 years, then decline across the rest of the reproductive lifespan. In an unselected population, circulating AMH concentrations were correlated with primordial follicle counts.^48^ Despite this, in a clinical setting, biomarkers of ovarian reserve including AMH concentrations and antral follicle count do not predict the clinically relevant outcomes of fertility, infertility or fecundity in the general population.^49^, ^50^, ^51^

While the validity of measuring AMH to predict the fertile lifespan and POI in women remains an ongoing source of investigation,^52^ our data in the present study add weight to the argument that AMH is not a reliable marker of the ovarian reserve on an individual, rather than a population level. We failed to find a correlation between primordial follicle number and serum AMH levels in a small cohort of human *BRCA1/2* mutation carriers, and furthermore, AMH levels were not reflective of significantly reduced ovarian reserve in *Brca1* cKO mice compared to WT at PN300. Notably though, an important limitation to consider in the present study is the fact that we did not have access to a matched cohort of human ovarian cortex and serum from a non-mutation carrier group. Therefore, this would be of interest to compare with mutation carriers in future studies.

In this study, we assessed fertility of mice lacking *Brca1* in oocytes in the absence of any exogenous, DNA damaging insults or challenge. Interestingly, even without an insult, we still detected higher levels of endogenous DNA damage in primordial follicles from *Brca1* cKO animals compared to WT controls at PN50 (reproductive age; peak fertility). Due to their increased propensity to develop cancer, *BRCA* mutation carriers are more likely than non-mutation carriers to be exposed to gonadotoxic anti-cancer therapies, which can act by inducing DNA DSBs.^4^ Therefore, analysis of the ovotoxic burden of chemotherapies, particularly in the context of BRCA deficiency, should be prioritised in future studies. As the landscape of cancer therapies evolves, new-line therapies such as PARP inhibitors and immunotherapies, including immune checkpoint inhibitors, are being administered to women, including for the treatment of triple negative breast cancer. Some evidence already suggests that PARP inhibitors^53^^,^^54^ and immune checkpoint inhibitors^55^^,^^56^ deplete ovarian follicles in WT mice. Testing the effects of these drugs in available preclinical models should be the subject of ongoing investigations and would also form an ideal platform to better study the precise DNA repair machinery recruited at lesion sites in the presence and absence of BRCA1.

Furthermore, in addition to its’ role in homologous recombination DNA repair, BRCA1 is a downstream component of the Fanconi anaemia pathway responsible for inter-strand crosslink repair and replication stress response.^57^ This pathway is likely to be important in key somatic cell types in the ovary undergoing rapid replication, such as granulosa cells. It would therefore be of interest to assess the ovarian phenotype of mice using a promoter such as *Foxl2*^58^ to conditionally delete *Brca1* in granulosa cells. This could lead to assessments of the function of BRCA1 in ovarian somatic cells across ageing and in response to genotoxic insults, such as anti-cancer agents.

To conclude, we correlated human primordial follicle counts and density in human ovarian cortical tissues with serum AMH concentrations from *BRCA1/2* gene mutation carriers. While our data showed no correlations, these results should be interpreted with caution due to the small sample size and advocate for further studies in larger cohorts. We also established a conditional knockout mouse model to study the contribution of BRCA1 in maintaining postnatal oocyte number and quality across the lifespan. Using this model, we demonstrated that conditional loss of *Brca1* in oocytes leads to reduced litter size, an age-dependent decline in the ovarian reserve of primordial follicles, and reduced oocyte quality which impairs production mature MII oocytes required for fertilisation. Given the critical role of the BRCA proteins in mediating DNA repair, our data extend the current knowledge of the quality control mechanisms in the female germline, in both primordial and mature oocytes and lay the foundations for future studies to test the specific effects of genotoxic insults on oocytes in context of loss of BRCA1. This information will improve understanding of the impacts to the fertility and long-term health of female cancer survivors, and techniques in fertility preservation counselling.^59^

## Contributors

KJH and ALW conceived and designed the study. ALW, LRA, JMS, YML, LT, EOKS, SG, YC, XC, MJG, NZ performed experiments. MH collected the WHAM cohort of human samples. ALW, LRA, YML, LT, EOKS, LG and KJH analysed and interpreted the data. ALW and LRA accessed and verified the underlying data. ALW wrote the manuscript. All authors read, edited and approved the final version of the manuscript.

## Data sharing statement

All data collected for this study is presented within this manuscript, and available to others on request from the corresponding authors.

## Declaration of interests

LG is a consumer advocate for Breast Cancer Trials (BCT), a consumer representative for Breast Cancer Network Australia (BCNA), a patient partner for Breast International Group (BIG), and a patient advocate for Roche (Roche Holding AG). All other authors declare no competing financial, or other interests.

## Appendix A Supplementary data

Supplementary Figure S1 **(a)** Schematic representation of the generation of WT (*Brca1*^*-/-*^*Gdf9*^*+/+*^) and oocyte conditional *Brca1* knockout (cKO: *Brca1*^*-/-*^*Gdf9*^*cre/+*^) female mice.

Supplementary Figure S2 Schematic depicting the validation of *Brca1* conditional gene deletion in mouse oocytes. **(a)** The *Gdf9-Cre* system with two *loxP* sites located on *Brca1* intron 11 and intron 12 was applied to conditionally knock out the functional exon of *Brca1* (exon 11) in oocytes. **(b)** Schematic of the generation of heterozygote fetal tissues for genotyping (*Brca1*^Δ11/+^). **(c)** Genotyping and sequencing information for detecting the truncated *Brca1* gene in *Brca1*^Δ11/+^ offspring. The *SnapGene* analysed results showed the presence of *loxP* (blue arrow) and the absence of *Brca1* exon 11 (red highlighted dash) in *Brca1*^Δ11/+^ fetal tail DNA when amplifying the region between *Brca1* intron 11 and intron 12 (PCR product length: 591 bp).

Supplementary Figure S3 **(a)** Full blot of genotyping gel result detecting the truncated *Brca1* exon 11, in every female offspring derived across three independent litters from different dams. **(b)** Annotated genotyping gel result. **(c)** Quantification of the proportion of offspring with the truncated *Brca1* exon 11 dependent on maternal genotype (n = 3).

Supplementary Figure S4 Analysis of all litters from each animal from the breeding trial was performed for WT and cKO females after continuous rounds of mating with proven C57BL6/J WT male studs. **(a)** Time taken to each litter and **(b)** the number of pups per litter, across all litters were recorded. Data are mean ± SEM; unpaired t-test; ∗p < 0.05.

Supplementary Figure S5 **(a)** Proportions of ovulated oocytes harvested following exogenous hormonal stimulation from WT and *Brca1* cKO mice at PN80 (WT n = 11; *Brca1* cKO n = 15) and **(b)** PN200 (WT n=8; *Brca1* cKO n = 6). Data are presented as mean ± SEM; unpaired t-test**.**

Supplementary Figure S6 Human cortical ovarian tissue sections from *BRCA1* (n = 8) and *BRCA2* (n = 10) mutation carriers (mc) were immunohistochemically stained for DDX4 as a marker of oocytes. **(a)** Primordial follicle and **(b)** total healthy follicle density per tissue volume (μm^3^) were calculated. Matched sample serum AMH concentrations (ng/mL) (dependant variable) were measured and correlated by Spearman’s rank correlation test with independent variables **(c)** total primordial follicle number, **(d)** total growing follicle number, **(e)** primordial follicle density per tissue area (μm^2^), **(f)** primordial follicle density per tissue volume (μm^3^) and **(g)** healthy total follicle number.
