# Supplementary figures and images for: Conditional loss of Brca1 in oocytes causes reduced litter size, ovarian reserve depletion and impaired oocyte in vitro maturation with advanced reproductive age in mice
Source: eBioMedicine. 2024 Jul 30;106:105262. doi: 10.1016/j.ebiom.2024.105262 (PMC11342213; doi:10.1016/j.ebiom.2024.105262)

SUPPLEMENTARY FIGURE 1

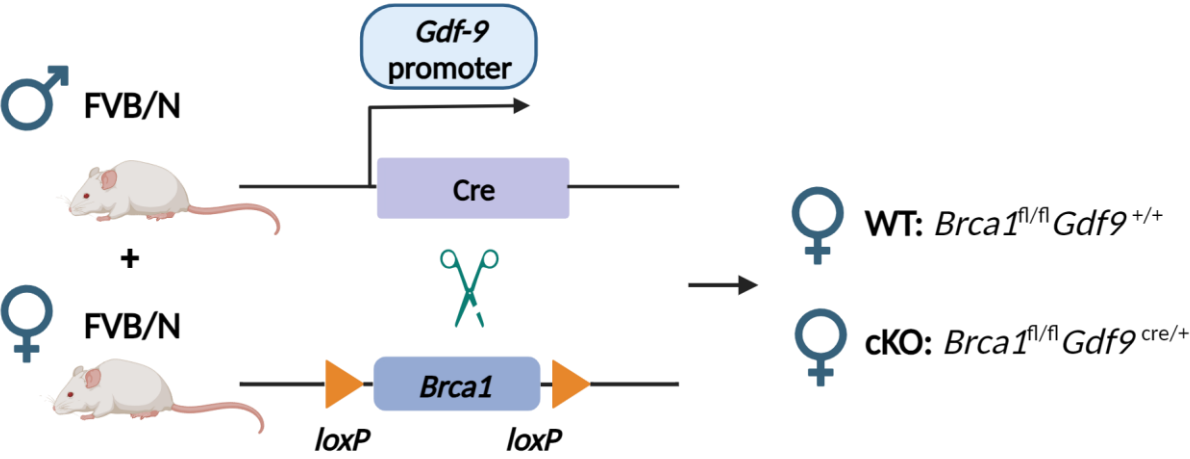

Supplement: Supplementary Figure S1 — (a) Schematic representation of the generation of WT (Brca1-/-Gdf9+/+) and oocyte conditional Brca1 knockout (cKO: Brca1-/-Gdf9cre/+) female mice. [file mmc1.pdf]

# SUPPLEMENTARY FIGURE 2

a

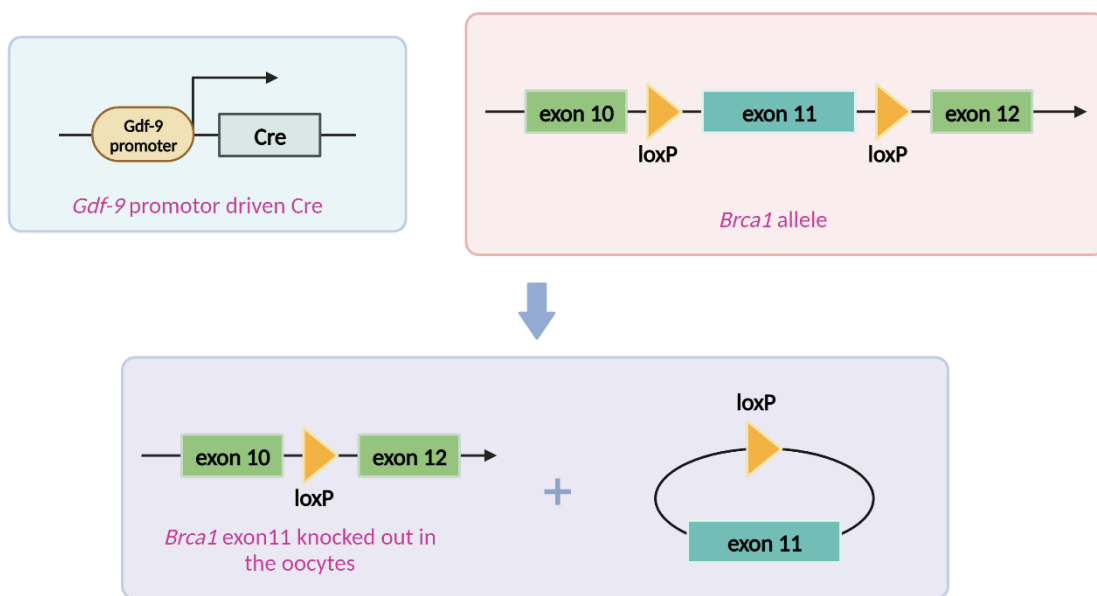

b

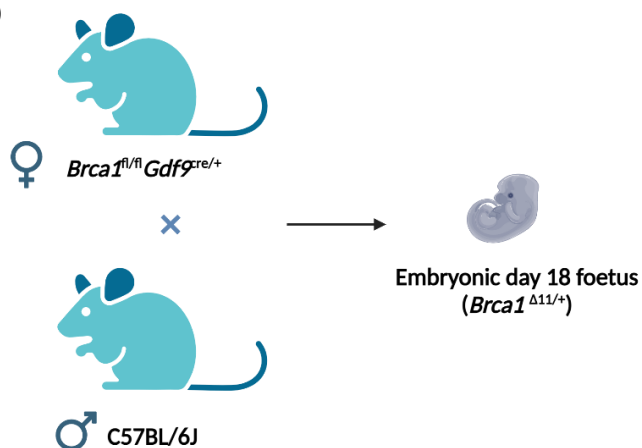

c

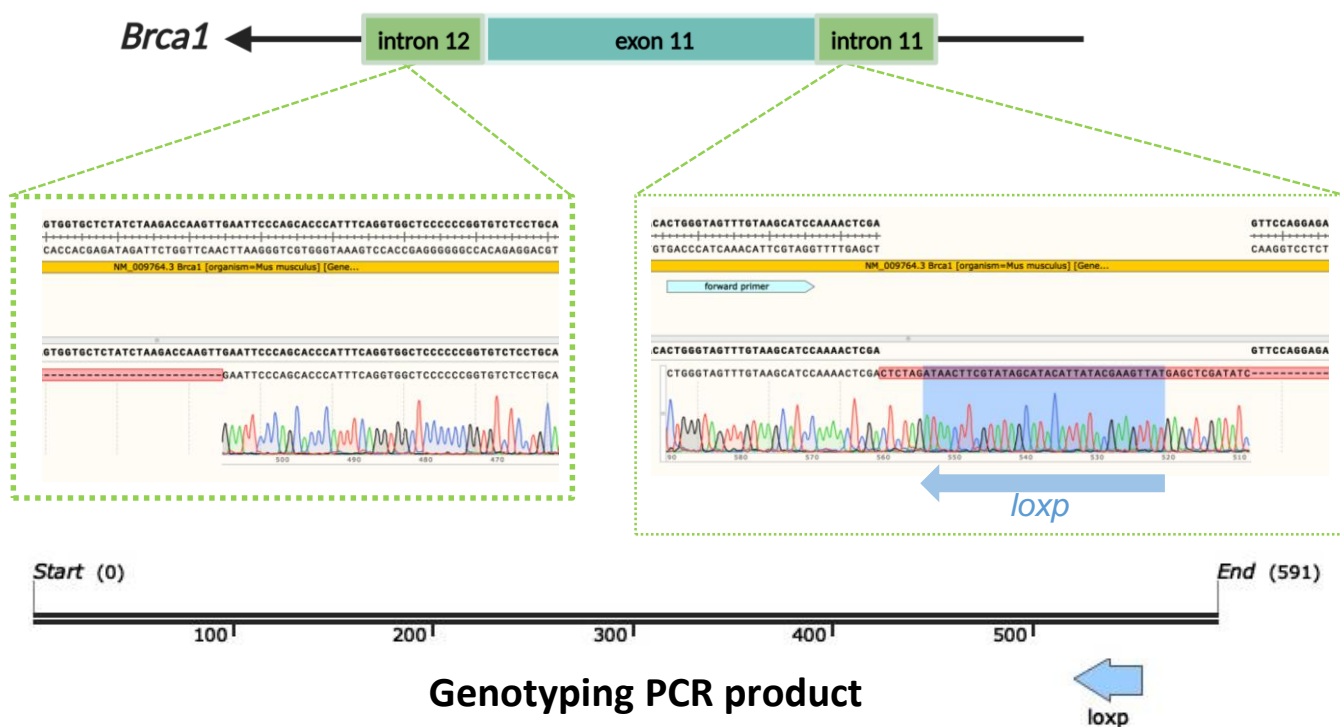

Supplement: Supplementary Figure S2 — Schematic depicting the validation of Brca1 conditional gene deletion in mouse oocytes. (a) The Gdf9-Cre system with two loxP sites located on Brca1 intron 11 and intron 12 was applied to conditionally knock out the functional exon of Brca1 (exon 11) in oocytes. (b) Schematic of the generation of heterozygote fetal tissues for genotyping (Brca1Δ11/+). (c) Genotyping and sequencing information for detecting the truncated Brca1 gene in Brca1Δ11/+ offspring. The SnapGene analysed results showed the presence of loxP (blue arrow) and the absence of Brca1 exon 11 (red highlighted dash) in Brca1Δ11/+ fetal tail DNA when amplifying the region between Brca1 intron 11 and intron 12 (PCR product length: 591 bp). [file mmc2.pdf]

# SUPPLEMENTARY FIGURE 3

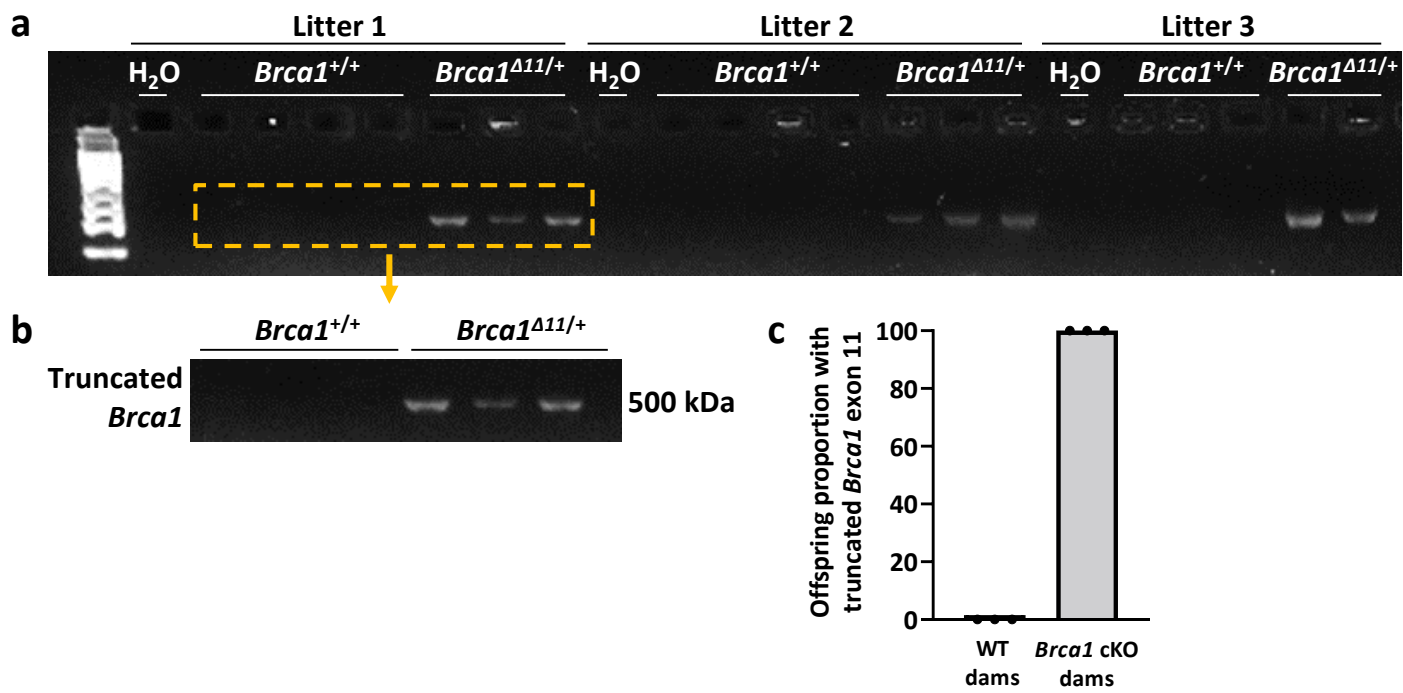

Supplement: Supplementary Figure S3 — (a) Full blot of genotyping gel result detecting the truncated Brca1 exon 11, in every female offspring derived across three independent litters from different dams. (b) Annotated genotyping gel result. (c) Quantification of the proportion of offspring with the truncated Brca1 exon 11 dependent on maternal genotype (n = 3). [file mmc3.pdf]

## a 2507

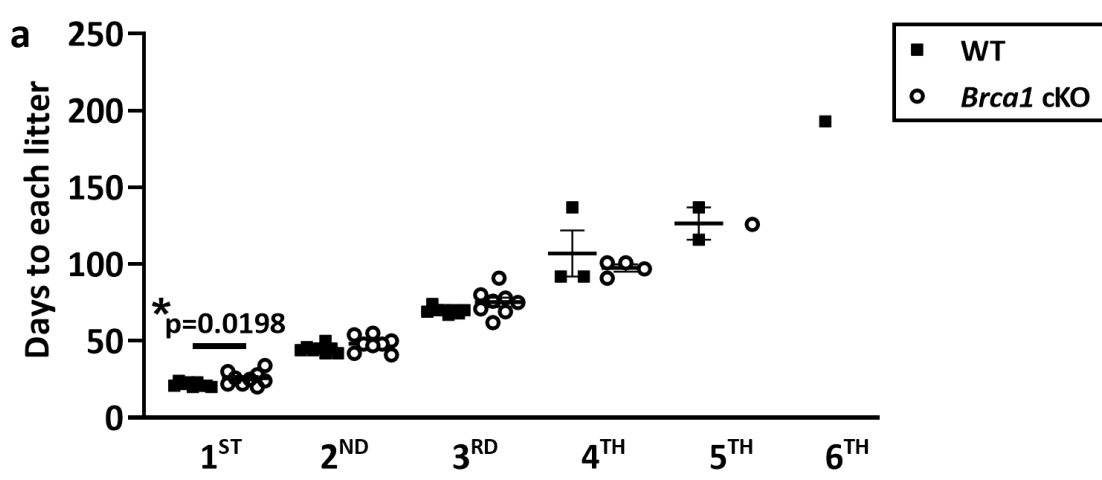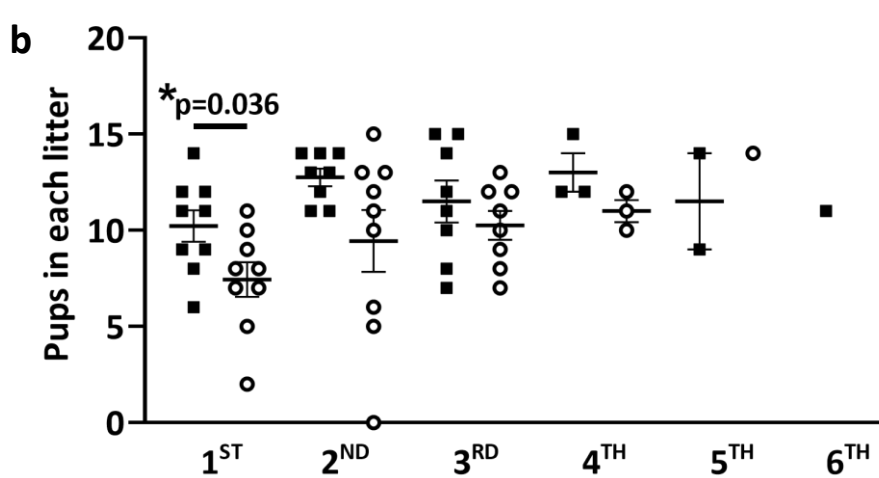

Supplement: Supplementary Figure S4 — Analysis of all litters from each animal from the breeding trial was performed for WT and cKO females after continuous rounds of mating with proven C57BL6/J WT male studs. (a) Time taken to each litter and (b) the number of pups per litter, across all litters were recorded. Data are mean ± SEM; unpaired t-test; ∗p < 0.05. [file mmc4.pdf]

SUPPLEMENTARY FIGURE 5

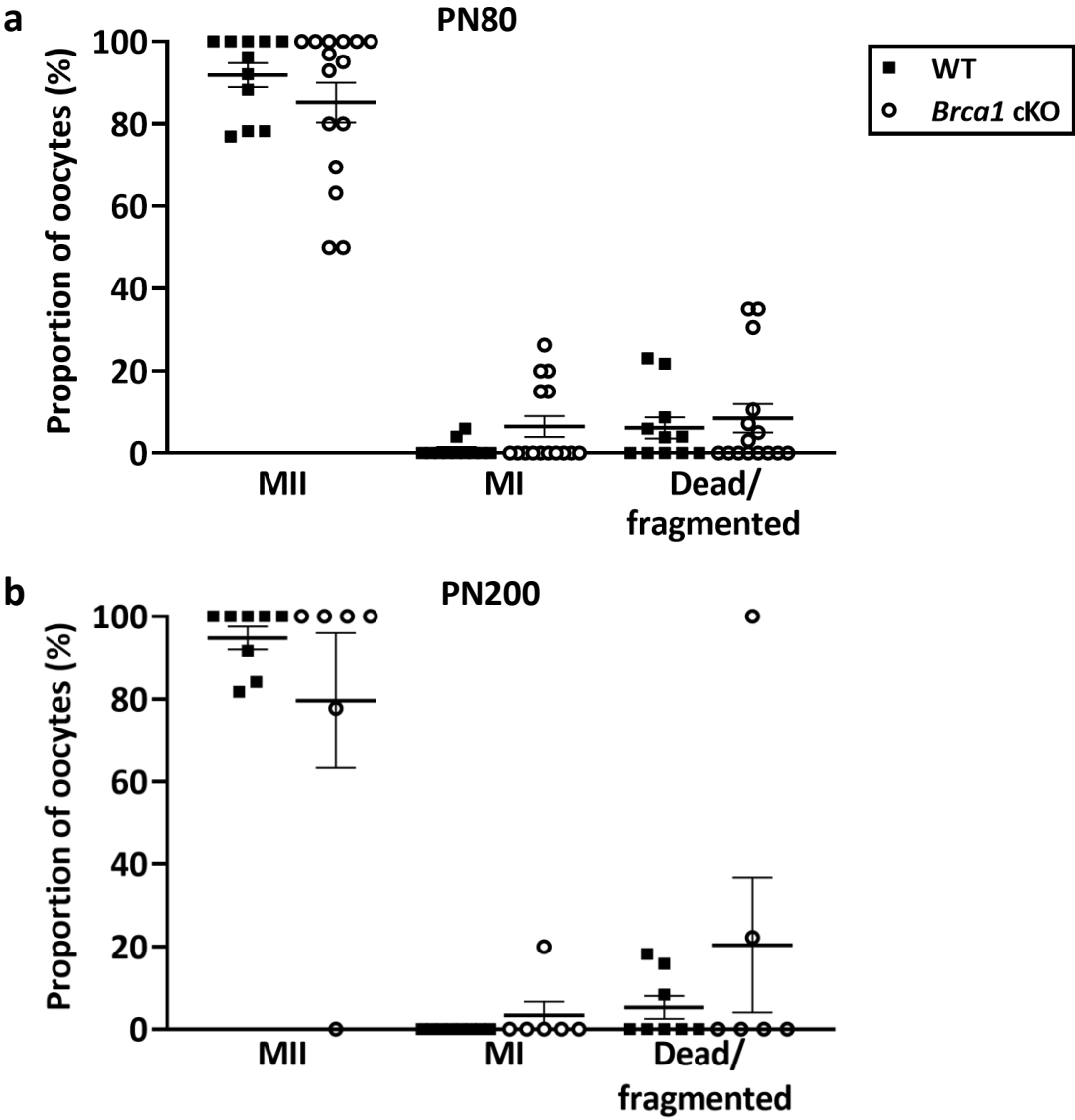

Supplement: Supplementary Figure S5 — (a) Proportions of ovulated oocytes harvested following exogenous hormonal stimulation from WT and Brca1 cKO mice at PN80 (WT n = 11; Brca1 cKO n = 15) and (b) PN200 (WT n=8; Brca1 cKO n = 6). Data are presented as mean ± SEM; unpaired t-test. [file mmc5.pdf]

SUPPLEMENTARY FIGURE 6

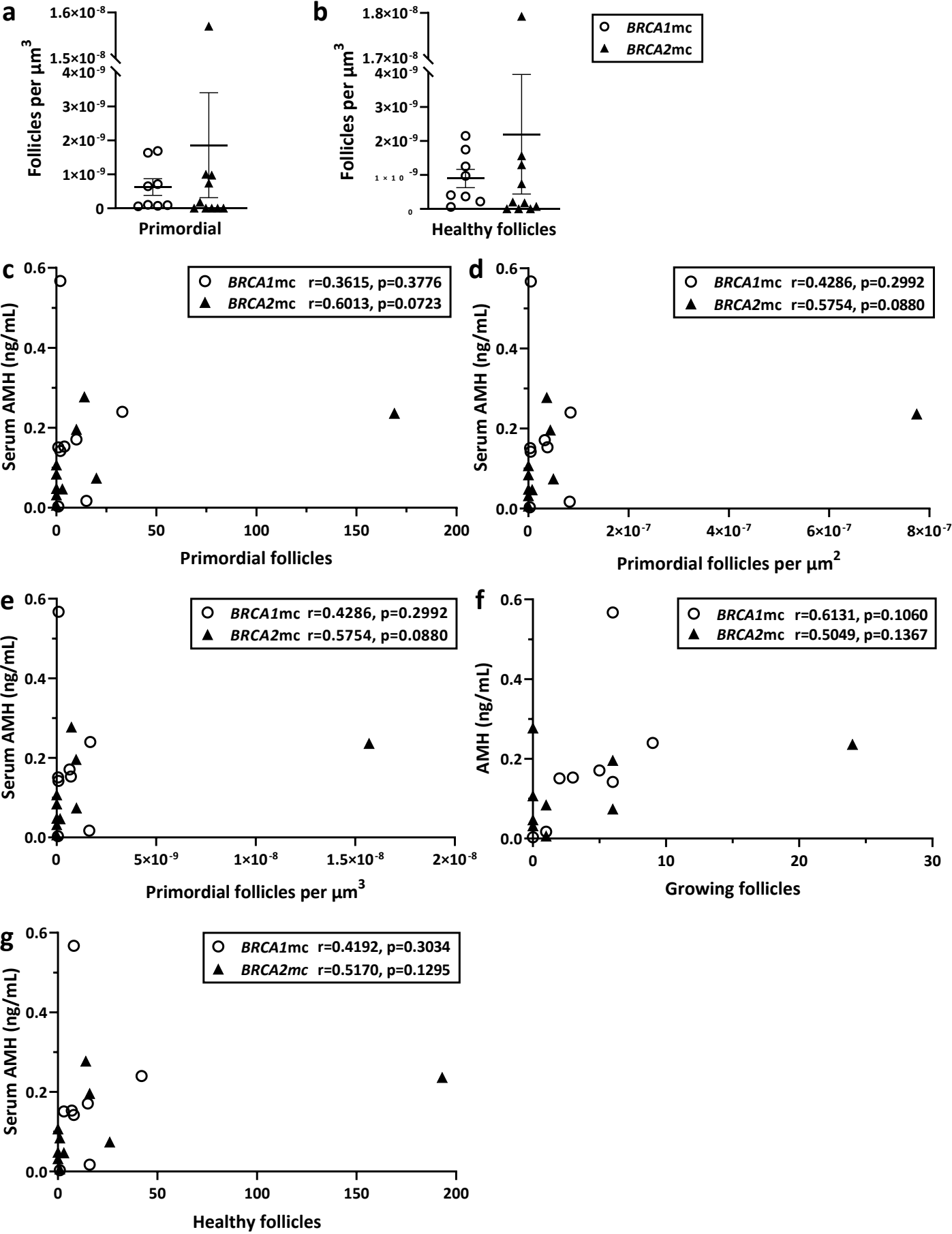

Supplement: Supplementary Figure S6 — Human cortical ovarian tissue sections from BRCA1 (n = 8) and BRCA2 (n = 10) mutation carriers (mc) were immunohistochemically stained for DDX4 as a marker of oocytes. (a) Primordial follicle and (b) total healthy follicle density per tissue volume (μm3) were calculated. Matched sample serum AMH concentrations (ng/mL) (dependant variable) were measured and correlated by Spearman’s rank correlation test with independent variables (c) total primordial follicle number, (d) total growing follicle number, (e) primordial follicle density per tissue area (μm2), (f) primordial follicle density per tissue volume (μm3) and (g) healthy total follicle number. [file mmc6.pdf]
